# The Maritime SPOR SUPPORT Unit (MSSU) Bridge Process: An Integrated Knowledge Translation Approach to Address Priority Health Issues and Increase Collaborative Research in Nova Scotia, Canada

**DOI:** 10.34172/ijhpm.2023.6901

**Published:** 2023-02-14

**Authors:** Julia Kontak, Amy Grant, Elizabeth Jeffers, Leah Boulos, Juanna Ricketts, Michael Davies, Marina Hamilton, Jill A. Hayden

**Affiliations:** ^1^Maritime SPOR SUPPORT Unit, Research and Innovation, Nova Scotia Health, Halifax, NS, Canada.; ^2^Nova Scotia Department of Health and Wellness, Halifax, NS, Canada.; ^3^Department of Community Health & Epidemiology, Faculty of Medicine, Dalhousie University, Halifax, NS, Canada.

**Keywords:** Integrated Knowledge Translation, Research Partnerships, Knowledge Broker, Priority Setting, Canada, Nova Scotia

## Abstract

**Background:** There is evidence of the benefits of integrated knowledge translation (IKT), yet there is limited research outlining the purpose of a knowledge broker (KB) within this approach. The Maritime SPOR SUPPORT Unit (MSSU) acts as a KB to support patient-oriented research across the Maritime provinces in Canada. The "Bridge Process" was developed by the Nova Scotia (NS) site as a strategy that involves work leading up to and following the Bridge Event. The process supports research addressing priority health topics discussed at the event by stakeholder groups. The objectives of this paper were to (1) describe the outputs/outcomes of this IKT approach; and (2) examine the role of the KB.

**Methods:** Quantitative data were collected from registration and evaluation surveys. Outputs are described with descriptive statistics. Qualitative data were collected through evaluation surveys and internal documents. Data related to KB tasks were categorized into three domains: (1) Knowledge Manager, (2) Linkage and Exchange Agent, and (3) Capacity Developer.

**Results:** The Bridge Process was implemented four times. A total of 314 participants including government, health, patient/citizen, community, and research personnel attended the events. We identified 24 priority topics, with 7 led by teams receiving support to complete related projects. Participants reported improved understanding of the research gaps and policy needs and engaged with individuals they would not have otherwise. Although patients/citizens attended each Bridge Event, only 61% of participants who completed an evaluation survey indicated that they were ‘actively engaged in group discussion.’ The KB’s role was identified in all three domains including Knowledge Manager (eg, defining questions), Linkage and Exchange Agent (eg, engaging stakeholders), and Capacity Builder (eg, research interpretation).

**Conclusion:** The MSSU facilitated an IKT approach by acting as a KB throughout the Bridge Process. This deliberative and sequential process served as an effective strategy to increase collaborative health research in the province.

## Background

**Key Messages**

** Implications for policy makers**

- Creating a deliberative and collaborative process to identify priority health research topics served as a mechanism to build relationships between different stakeholders across the health system.
- The process enabled the formation of new collaborative partnerships to bridge the gap between research, policy, and practice.
- Having an organization dedicated to serving as a knowledge broker (KB), with appropriate research supports in place, helps to facilitate the connections between stakeholder groups and the movement of evidence into policy and practice.
- Collaborative discussion of health topics allows for tailored identification of knowledge gaps that consider perspectives across policy, research, practice, and from the perspective of patients/community partners.

** Implications for the public**

The Maritime SPOR SUPPORT Unit (MSSU), a local research support organization, developed and implemented a process called the “Bridge Event.” This event brings together researchers, policy-makers, clinicians, and patients/citizens to identify health topics of the highest priority – as in, health system issues that need to be addressed right away. By bringing together these different groups, the MSSU aims to ensure that the views of patient/citizen partners are incorporated into the research process, and that individuals who are going to enact policy changes as a result of the research findings are present to ensure the questions are of priority and that changes can be implemented when the research is complete.

There is widespread acknowledgement of the distinct gap, often referred to as the “know-do gap,” which represents the divide between the evidence produced in health research and what is used in healthcare decision making.^1-3^ Historically, this gap was presumed to be a result of research being packaged inaccessibly to users of the evidence (ie, knowledge users), yet it is now recognized that intentional tactics need to be used for research to be useful, more actionable, and embedded.^1,4^ To do so, a growing focus is being put on integrated knowledge translation (IKT), an approach to facilitate the movement of evidence into practice by engaging knowledge users throughout each step of the research process.^2,5,6^ The purpose of this approach is to ensure knowledge users’ expertise and perspectives are considered throughout the process^4,6-8^ in order to produce research that is more context-relevant and useful in supporting healthcare decision-making.^1,9,10^ IKT draws on similar methods to participatory research approaches including co-creating knowledge with knowledge users and acknowledging varying perspectives strengthen rather than devalue the research process; yet the purpose of the approaches differ.^6^ Participatory approaches aim to identify community-driven solutions, while IKT approaches engage knowledge user expertise in research-informed strategies to ensure the work is appropriate and of use in a policy and practice setting.^6^ That said, benefits of IKT are well established, including more timely and relevant research,^1,11-13^ greater credibility of the work produced,^14,15^ enhanced capacity for researchers and knowledge-users involved,^15^ increased opportunities for collaboration and networking across stakeholder groups,^15,16^ and rebalancing power dynamics by challenging what is considered “expertise.”^2^ Despite the collaborative benefits of IKT approaches, there is a dearth of literature outlining the practical components that are involved in the development, coordination, and management of this approach,^9,15,17^ as well associated long-term outcomes.^1,18,19^ Gagliardi and colleagues’^9^ scoping review on IKT in healthcare included an overview of IKT approaches with meetings (eg, conferences, presentations, workshops) identified as the most frequent, but the nature and detail of the activities varied in scope. Due to the diversity in contextual factors within IKT approaches, strategies for positive outcomes remain unknown.^9^ The limited knowledge on IKT approaches makes it difficult to systematically understand and replicate the actionable steps of an IKT approach, as well as measure the outcomes over time.

 An actionable component of the IKT approach that is becoming increasingly common is the role of knowledge brokers (KBs). KBs, a term used to define the human component of knowledge translation (KT) strategies, can take shape as individuals or organizations who are considered vital in supporting the coordination and management of IKT, and contribute to the sustainability of collaborative research partnerships.^20^ KBs are often referred to as “connectors” or “intermediators,” and are increasingly viewed as playing a vital role in facilitating the iterative process of closing the “know-do-gap.”^20,21^

 In order to facilitate IKT partnerships that meet the research, policy and practice needs of local stakeholder groups, our team—the Maritime Strategy for Patient Oriented Research (SPOR) SUPPORT (Support for People and Patient-Oriented Research and Trials) Unit (collectively referred to as the MSSU)—has taken on the role of a KB by supporting the production and implementation of patient-oriented research across the Maritime provinces (Nova Scotia [NS], New Brunswick and Prince Edward Island) in Canada. The MSSU works as a connector between key stakeholder groups by collaborating with patient/citizen partners, governmental departments, health authorities, and the research community, within and across the Maritimes to ensure diverse perspectives are integrated into the research process. The MSSU adopts the Canadian Institutes of Health Research (CIHR) definition for patient and citizen. A patient is “an overarching term that includes individuals with personal experience of a health issue and informal caregivers, including family and friends,*” *and a citizen “encompasses interested representatives of the general public, consumers of health services, patients, caregivers, advocates and representatives from affected community and voluntary health organizations.”^22^ The MSSU is one of 11 SUPPORT Units across Canada that form an integral part of the SPOR initiative funded by CIHR. Each location has built unique mechanisms to help facilitate the movement of evidence into policy and practice.

 To facilitate evidence-informed decision-making in NS, we developed an IKT approach to identify and support research, now known as the Bridge Process. The Bridge Process is an overall strategy, which focuses on work leading up to and following an event called the Bridge Event. We developed five main steps to identify and then support research addressing priority health topics in the province, which are initially discussed at the Bridge Event. The Bridge Event is a day-of knowledge exchange event that brings together multidisciplinary stakeholder groups to discuss priority health issues, identify knowledge gaps, and potential research projects related to the topics. Given the scarcity of literature providing practical steps to implement an IKT approach, or that speaks to the research, policy, and practice outcomes of IKT, we seek to report on our Bridge Process approach in the current study to contribute to this body of knowledge. The objectives of the current research are to (1) describe the methods used by the MSSU to implement the Bridge Process, and to report on the outputs and outcomes of these strategies, and to (2) examine the associated tasks and function of the MSSU as a KB within the Bridge Process.

## Methods

###  MSSU Bridge Process

 The Bridge Process was developed at the MSSU NS site to facilitate local interdisciplinary, collaborative research partnerships to address priority health issues in the province. This process includes five stages to integrate knowledge user expertise and perspective into the research process, including a day-of knowledge exchange event titled the Bridge Event. The goals of the Bridge Process are two-fold: (1) Build connections and collaborations across health system stakeholder groups by providing a forum to discuss priority health topics from diverse perspectives, and (2) Identify knowledge gaps in policy and practice where research evidence could help to inform practice changes, health system changes, and/or policy decisions.

###  Stage 1: Identify and Prioritize Research Questions

 The approach to solicit priority health topics was guided by the Contextualized Health Research Synthesis Program developed by the Newfoundland and Labrador Centre for Applied Health Research^23,24^ and follows a stepwise process outlined in Figure. The steps include the following: (1) MSSU stakeholder engagement meetings, (2) MSSU topic review, (3) Stakeholder research topic review, (4) Prioritization of questions, and (5) Select Bridge Event research questions.

**Figure:**
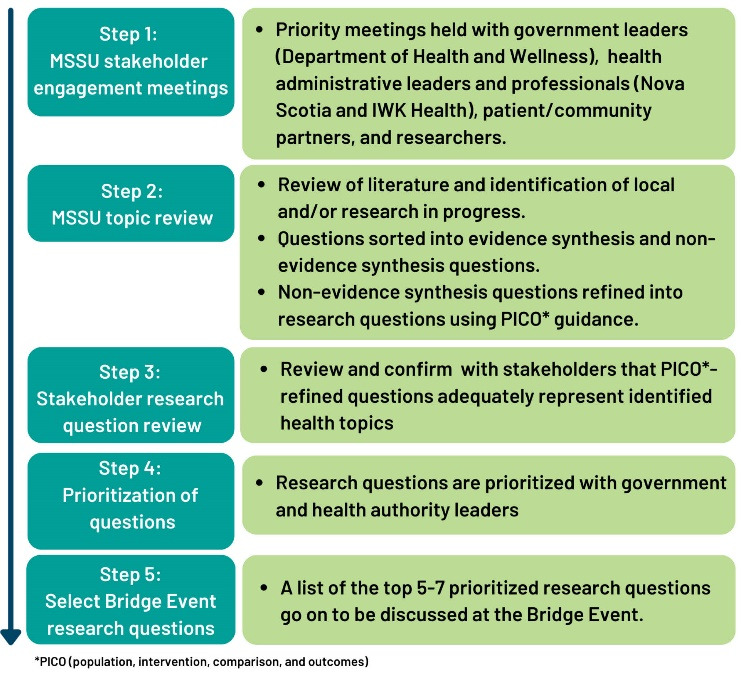
Priority Topic Identification Process. Abbreviation: MSSU, Maritime SPOR SUPPORT Unit.

 In Step 1, The MSSU team collaborates with the two provincial health authorities (NS Health and IWK Health), and the NS Department of Health and Wellness, to identify pressing health topics relevant to health service delivery, policy, and/or the health needs of the population. Additionally, researchers, and community stakeholders (eg, community groups, research organizations, members of the public, patient/citizen partners) may be invited to identify priority topics or to further refine priority topics if they are closely connected (through community work, lived experience, or research interest/expertise) to a topic identified by the Department of Health and Wellness or the provincial health authorities.

 In Step 2, the MSSU reviews topics and consolidates the list as outlined in Figure. Questions are divided into evidence synthesis and non-evidence synthesis questions, the latter of which are appropriate for discussion at the Bridge Event.

 In Step 3, this consolidated list returns to stakeholders who identified the initial research questions, to ensure the topics are still relevant and represent the issues identified. In Steps 4 and 5, these questions are then prioritized with government and health authority leadership, such that we can narrow down the list to a maximum of 7 topics that can be discussed at the Bridge Event. The number of topics chosen is based on very practical reasons – which include the maximum room capacity for participants at the event and capacity for the MSSU to support research teams who go on to request support.

###  Stage 2: Engage Stakeholders to Build Collaboration

 Once priority health research questions are identified, the MSSU NS site utilizes its province-wide networks to engage government decision-makers, health professionals, researchers, and patient/citizen partners with relevant expertise and interest in these priority areas. Through our collaboration with other networks and leaders within the health authorities, we identify researchers with expertise in the areas identified, and decision-makers who are appropriate to the topic identified based on their role within the institution. We also reach out to researchers and community partners by broad searches on the internet, and on university websites using keywords from the topics of interest. Patient/citizen partners are identified through the MSSU Patient Engagement Coordinator, who regularly communicates with a group of individuals who work on MSSU research and committees. Other patients/citizens may be identified from clinicians, researchers, or community organizations who have knowledge of potential patients/citizens who have lived experience in the topic area. Identified individuals with diverse perspectives and expertise related to the priority health topics are invited to attend the Bridge Event. The Bridge Event is also more widely communicated and open for registration through newsletters to the health research community (which includes clinicians, researchers, patient/citizen partners affiliated with the health authorities or MSSU). Given the nature of the event, there are limitations on attendance based on availability, staffing changes, and interest. Thus, after the event, if the team discussing the topic feels other perspectives are needed, or identifies individuals (clinicians, policy-makers, etc) who are a good fit for the topic area, those additional individuals will be identified and invited to join the team.

###  Stage 3: Identification of Knowledge Gaps

 Prior to the event, each individual attending receives a topic-specific evidence brief for the topic they have registered to discuss. On the day of the event, key stakeholders are assigned to teams to engage in discussion on the assigned topic. Each team works together on one topic to identify knowledge gaps and potential research projects to address the topic and associated issue(s). A facilitator is assigned to each team, and is provided with a guiding document (Supplementary file 1) to take them through semi-structured exercises to narrow the focus, clarify the research question, and ultimately identify a feasible research question to address the priority topic. At the end of the event, each team summarizes the discussion their group had with all the event participants and identifies any potential research directions for the topic discussion.

 If the refined research question aligns with the needs and interests of the team (which includes decision-makers, researchers and patient/citizen partners) who discussed the topic at the event, the MSSU supports further discussion of the research question at follow-up meetings after the Bridge Event.

###  Stage 4: Developing and Fostering Collaborative Research Partnerships

 Following the Bridge Event, the MSSU supports the newly formed teams by providing discussion summaries and suggestions for next steps, facilitating continued group interactions (eg, supporting follow-up meeting organization and team communication), making connections to potential new team members not already engaged, and providing research support for the further refinement of questions and project ideas. The MSSU’s primary role at this stage is to foster interactions to enhance the early stages of collaborative research partnerships. Teams that decide to initiate a research project go forward to Stage 5. However, if a team decides they do not want to pursue a research project, but identify other opportunities related to the priority topic (eg, training session or workshop), the MSSU may provide support via these different avenues.

###  Stage 5: MSSU Priority Project Application and Support

 Teams that decide to initiate a research project based on a refined research question can apply for MSSU support through an MSSU Priority Project application process (Supplementary file 2). MSSU Priority Project teams receive access to in-kind project management and research supports which may include evidence synthesis expertise, patient engagement, methods support, administrative health data navigation, data analysis, and KT, teams that apply for MSSU Priority Project support must identify science, decision-maker and patient/citizen partner leads and outline how the research will be used for practice and policy decision-making. Project support is approved through a Provincial Steering Committee of government, health authority, research, clinical, and patient/citizen partner representatives who provide stewardship to the MSSU to enhance patient-oriented research and evidence-informed outcomes in each province.

###  Data Collection

 An online evaluation survey for each Bridge Event (Stage 3) was developed and administered through Select Survey v4.1 to all attendees following the events. Evaluation of the partnership formed and research process is evaluated for Priority Projects once complete and will not be reported in this current manuscript. The evaluation survey included questions related to event organization (eg, facilitation, supplementary documents provided) and event objectives (networking, evidence gaps, policy needs, and patient and community engagement) (Supplementary file 3). Close-ended questions around satisfaction with the event were evaluated on a 5-point Likert scale ranging from Strongly Disagree to Strongly Agree. Open-ended questions related to future recommendations and general comments were also included. Relevant data was exported into Microsoft Excel for analysis. Descriptive data related to the number and type of role that best describes a participant was collected through our registration platform to the Bridge Event. Information related to the number and type of priority topics identified, refined, and approved was tracked separately in Microsoft Excel.

 Qualitative data of the MSSU Priority Projects were also collected through document review of the MSSU’s annual reports from 2018-2019 fiscal year to 2020-2021 fiscal year, MSSU’s 2019-2020 report to community, and internal project summary and tracking. These documents outline in detail key achievements, impacts and processes of the unit’s work over each year. Information related to the Bridge Process, associated MSSU Priority Projects, collaborations and processes were retrieved for analysis.

###  Data Analysis

 We described results of the evaluation survey, and information related to the identification, prioritization, and project outputs that resulted from the Bridge Process using counts, frequencies and percentages, carried out using Microsoft Excel. Responses to open-ended questions were analysed using direct content analysis^25^ and grouped into the categories of patients, policy, collaboration, topic, and organization. Narrative description of MSSU Priority Projects and outcomes are also categorized into the two main aims of the Bridge Process: (1) Build connections and collaborations across health system stakeholder groups by providing a forum to discuss priority health topics from diverse perspectives, and; (2) Identify knowledge gaps in policy and practice where research evidence could help to inform practice changes, health system changes, and/or policy decisions.

 Tasks and functions executed by the MSSU staff throughout the Bridge Process were categorized by an individual researcher (JK) in Microsoft Excel using the guiding definition of a KB and domains outlined by Bornbaum et al.^20,21^ One researcher (AG) reviewed the categorizations, and any discrepancies were discussed and resolved through consensus. The three domains used for categorization included “Knowledge Manager,” “Linkage and Exchange Agent,” and “Capacity Builder.” Each domain outlines supplementary tasks that are either independent to one domain (ie, Knowledge Manager) or overlap with two or more domains (ie, Knowledge Manager and Linkage and Exchange Agent). For ease of analysis, each supplementary task is only displayed by their primary domain and only tasks relevant to the Bridge Process were included.

## Results

###  MSSU Priority Projects Outputs and Outcomes (Objective 1)

 The Bridge Process occurred four times (in June and November of both 2018 and 2019). A total of 314 participants attended Bridge Events including representatives from government (n = 33), health authorities (n = 109), patient/citizen partners (n = 35), research/universities (n = 123) and other organizations (n = 14) (Table 1). As outlined in Table 2, the Bridge Process has identified 66 priority topics with 24 topics being refined into research questions and discussed at one of the four events. A total of seven priority topics went on to receive MSSU Priority Project support and two priority topics became spin-off projects (eg, workshops, collaborations). The title of each MSSU Priority Project, general area of focus, stage and current outputs are outlined in Table 3. Further information related to the description of each MSSU Priority Project, and links to outputs are included in Supplementary file 4.

**Table 1:** Bridge Event participation

**Participants **	**Bridge Event 1**	**Bridge Event 2**	**Bridge Event 3**	**Bridge Event 4**	**Total **
Government	14	9	7	3	33
Health authorities	24	25	47	13	109
Patient/citizen partners	9	8	7	11	35
Research/University	41	34	19	29	123
Other organizations	3	5	2	4	14
Total	91	81	82	60	314

Note: Participant numbers per event are based off registration information and may be subject to (+/-) attendees due to absences or day-of attendance without registration that was not recorded.

**Table 2:** Bridge Process Topic Identification and Prioritization Summary

**Priority Topics **	**Bridge Process 1**	**Bridge Process 2**	**Bridge Process 3**	**Bridge Process 4**	**Total **
No. of priority topics refined	20	15	14	17	66
No. of evidence synthesis topics identified	0	6	12	5	23
No. MSSU Priority Project topics identified	7	5	7	5	24
No. of MSSU Priority Projects approved	3	1	2	1	7
No. of project spin-offs (eg, workshops or projects)	1	0	1	0	2

Abbreviation: MSSU, Maritime SPOR SUPPORT Unit.

**Table 3:** Bridge Process Priority Projects, Area of Focus, Stage and Current Outputs

**Project Title**		**Area of Focus**	**Stage**	**Deliverables**						
				**Plain Language Summary **	**Infographic**	**Report**	**Peer-reviewed Publication**	**Conference Presentation**	**Knowledge Exchange Presentation**	**Spin-off funding**
BP 1	UniCITY: Uniting to connect innovative technology for youth mental health and addictions services	Mental health and addictions	Part 1: Complete Part 2: Complete	X		X	X			X
	Barriers and enablers to implementing interprofessional collaborative family practice teams with a focus on improving access to primary care	Primary care	Phase 1: Complete Phase 2: In progress	X	X	X	X*	XX	XX*	X
	Current management and healthcare quality for patients with hip and knee osteoarthritis	Osteoarthritis	In progress	X*	X*	X*	X	XX		X
BP 2	Pharmacist prescribing and primary healthcare access	Pharmacy	Complete	X*	X*X*	X*	XX*	XXXX		X
BP 3	Youth and young adult vaping: gathering evidence and guiding practice	Public health	Complete		XX					
	Exploring the transition from pediatric to adult care	Transitions in care	Part 1: Complete Part 2: In progress	X	XXX	X	XX*	XXX	XXXXXXXX*	XX*X*
BP 4	How does NV best harness the assets of its extensive post-secondary educational system to improve regional health outcomes?	Learning health systems	Part 1: In progress Part 2: Not started					X	X	

Abbreviations: BP, Bridge Process; NS, Nova Scotia. Note: Number of X’s = number of products developed for each deliverable. Table up to date as of September 29, 2022. *= in preparation or submitted.

 In relation to the Bridge Event, evaluation data are presented by main activity and broken down by event in Table 4. The results are representative of participants who completed the post-event evaluation survey. In total, the completion rate across all Bridge Event’s was 42% (n = 131/314 total attendees). Broken down by event, completion rates were 33% (n = 30/91; Bridge Event 1), 44% (n = 36/81; Bridge Event 2), 44% (n = 36/82; Bridge Event 3), and 48% (n = 29/60; Bridge Event 4).

**Table 4:** Bridge Event Evaluation Survey Results

**Evaluation Question**	**Bridge Event 1 (n = 30)**			**Bridge Event 2 (n = 36)**			**Bridge Event 3 (n = 36)**			**Bridge Event 4 (n = 29)**		
	**Agree**	**Neutral**	**Disagree**	**Agree**	**Neutral**	**Disagree**	**Agree**	**Neutral**	**Disagree**	**Agree**	**Neutral**	**Disagree**
The evidence summary was useful for group discussion, No. (%)	N/A	N/A	N/A	26 (74)	6 (17)	3 (9)	N/A	N/A	N/A	12 (50)	8 (33)	4 (17)
The approach to the facilitated discussions was effective, as a means of identifying gaps and policy needs for a specific topic area, No. (%)	21 (70)	7 (23)	2 (7)	30 (83)	3 (8)	3 (8)	26 (74)	6 (17)	3 (9)	19 (79)	3 (13)	3 (8)
Patients/citizens were actively engaged and represented in the group discussion, No. (%)	N/A	N/A	N/A	24 (67)	7 (19)	5 (14)	23 (67)	4 (12)	7 (21)	11 (44)	7 (28)	7 (28)
There was enough time for networking with others during this event, No. (%)	N/A	N/A	N/A	29 (83)	4 (11)	2 (6)	27(77)	6 (17)	2 (6)	13 (52)	5 (20)	8 (32)
I have engaged with researchers, healthcare providers, decision-makers, and/or patients/citizens I otherwise would not have met, No. (%)	26 (86)	4 (14)	0 (0)	31 (91)	2 (6)	1 (3)	30 (88)	4 (12)	0 (0)	19 (79)	4 (17)	1 (4)
I have a greater understanding of the gaps and policy needs in the specific topic areas presented today, No. (%)	20 (69)	7 (24)	2 (7)	30 (85)	4 (11)	1 (3)	29 (85)	2 (6)	3 (9)	18 (75)	5 (21)	1 (4)

Abbreviation: NA, Not applicable. Note: Each question on the survey was not mandatory, thus the number of participants who responded to the survey and the number of individuals who answered each question may differ.

 On average, across all four events, 76% of participants agreed that the facilitated discussion was effective. Most participants (87%) also agreed that they had engaged with others they would not have otherwise met.

 In comparing responses for each event, there were differences in the extent to which participants agreed that they improved their understanding of the gaps and policy needs for the identified topics (69% at Bridge Event1, 85% at Bridge Event2 and 3, and 75% at Bridge Event4). Similarly, evaluation of patient/citizen engagement varied over the events. At Bridge Event4, only 44% of respondents agreed that patients/citizens were actively engaged and represented in the group discussion, whereas 67% agreed with this statement at Bridge Event2 and 3.

 A total of 99 (n = 23, 26, 32, 18 from Bridge Event1, 2, 3, and 4, respectively) respondents answered the open-ended questions around ‘additional topic areas or research questions’ or provided ‘general comments/feedback.’ The responses were grouped into five categories: patients, policy, collaboration, topic, and organization. There were 20 comments around topic, and 96 around organization which was primarily around suggestions for future topics and event logistics, and thus was used for internal planning and is not reported further here.

 A total of 16 respondents discussed patient engagement, which identified that there was a need to hear more from patient/citizen voices at the discussion tables. Some participants also identified that having patients/citizens who served multiple roles (eg, were also healthcare providers) was not ideal and that this did not represent ‘authentic’ perspectives for patients/citizens navigating the system. In contrast, some patients reported that they did not want to be forced to discuss just one aspect of their experience given that they work in multiple roles. Additionally, there were suggestions around the need to consider venues that are more accessible to patients/citizens – both physical accessibilities, but also comfort in accessing a location that patients/citizens feel accepted and welcomed (eg, outside an academic setting). In terms of policy-directed feedback, there were a total of 15 responses in this category, with respondents expressing the need for more policy and healthcare provider voices. There were 10 comments discussing collaboration, sharing appreciation for the opportunity to collaborate across stakeholder groups – bridging policy, practice, and lived experience.

###  Description of MSSU Priority Projects, Outputs and Outcomes 

 Beyond the evaluation survey outputs of each Bridge Process, there are wider benefits that are described below. Qualitative outcomes were classified according to the two main aims of the Bridge Process:

####  Bridge Aim 1: Build Connections and Collaborations Across Health System Stakeholder Groups by Providing a Forum to Discuss Priority Health Topics From Diverse Perspectives

 Connection and collaborations between stakeholder groups are intertwined throughout each stage of the Bridge Process. These relationships can take varying forms that may lead to collaborative research partnerships through the establishment of a MSSU Priority Project or can include linkages and collaborations that may not have had the opportunity to be formed otherwise. Teams initiating a MSSU Priority Project must identify a science, decision-maker, and patient/citizen partner lead to move forward with in-kind MSSU support, which lays the precedent for meaningful and sustained collaboration over-time.

 A current MSSU Priority Project titled *Exploring the transition from pediatric to adult care* (Table 2) is a noteworthy example of a collaborative research partnership developed through the Bridge Process. Patient/citizen partners, the Department of Health and Wellness, and healthcare professionals from IWK and NS Health have been involved in the study from its inception to implementation. Specifically, the decision-maker lead is the Transition Coordinator at IWK Health and provides the team with an invaluable practical perspective and linkage to the Transition of Care Committee. Further, the team includes four patient/citizen partners in addition to other healthcare stakeholders in the project who assist with the protocol, data collection tools, methods for data analysis, interpretation of findings and dissemination activities.

 Similarly, the MSSU Priority Project *Pharmacist prescribing and primary healthcare access* (Table 2) involves a strong collaboration between Dalhousie University College of Pharmacy, Department of Health and Wellness, Nova Scotia College of Pharmacists, Pharmacy Association of Nova Scotia, and NS Health. The foundation of this collaboration was developed at the Bridge Event, which established the partnership with a diverse set of stakeholder perspectives to ensure the research conducted is applicable to policy and practice.

 As previously mentioned, not all connections and collaborations made at Bridge Events develop into priority projects, but secondary outputs, often referred to as “spin-off” opportunities, may occur. For example, a priority topic identified and discussed during the Bridge Event 3 (June 2019) related to the implementation of the NS deemed consent policy for human organ and tissue donation, brought together established and new stakeholders. The Bridge Event introduced team members to a pre-existing group that had been recently successful in receiving Health Canada funding to carry out a project titled* Legislative evaluation: Assessment of deceased donation reform program. *The Bridge Event linked current team members to additional stakeholders including clinicians and patient/citizen partners. MSSU staff are now well-embedded within the team, helping to support different components of this research program including KT, patient engagement, data access and methodology support.

####  Bridge Aim 2: Identify Knowledge Gaps in Policy and Practice Where Research Evidence Could Help to Inform Practice Changes, Health System Changes, and/or Policy Decisions

 The Bridge Process provides a forum for stakeholders to connect and explore how research evidence can help to inform policy and practice. The development of collaborative research partnerships has had positive impacts on real-word initiatives. The MSSU Priority Project *UniCITY: Uniting to connect innovative technology for youth mental health and addictions services *(Table 2) was able to bring together team members that would not previously have had the opportunity to collaborate. Due to this collaboration, some team members went on to develop a text messaging intervention for youth and parents that is currently being implemented and tested at the IWK Health. An additional example of implication to policy and practice is a workshop that was carried out following Bridge Event1 (June 2018) that focused on sharing local research around opioid prescription from different prescribers (eg, dentists, physicians) across different settings (eg, emergency departments, family medicine clinics) and patient populations. The workshop brought together stakeholders from different areas of the health system, including researchers, clinicians, policy-makers, and health organizations involved in the funding or licensing of care providers.

 In addition to the supports already outlined, if a team identifies a knowledge gap that requires further research funding to help inform change, the MSSU will provide in-kind support to help apply for funding opportunities to support next steps in the research agenda. For example, an MSSU Priority Project team that completed a review of the literature on barriers and enablers to inter-professional collaborative family practice teams (Table 2), applied and was successful in receiving NS Health Translating Research into Care funding^26^ to further explore barriers and enablers within the provincial context.

 On a national scale, the *Pharmacist prescribing and primary healthcare access *project (Table 2) helped inform a subsequent successful application for the CIHR COVID-19 Rapid Research Operating Grant for a research study titled PUPPY^[1]^. This study seeks to understand the needs of patients and primary care providers before, during, and after coronavirus disease 2019 (COVID-19). This success was in part due to collaboration between these two project teams, showcasing the ability of the Bridge Process to mobilize efforts and foster collaboration across research groups to ensure the most extensive evidence is produced to support and inform healthcare decision-making.

###  Knowledge Broker Function (Objective 2)

 The roles and responsibilities of the MSSU as a KB throughout the Bridge Process are summarized in Table 5 and described below by the three previously identified KB domains.

**Table 5:** Knowledge Broker Domains, Tasks and Bridge Process Examples^20^

**Knowledge Management** ^20^: *Ability to coordinate and navigate the complex process of moving evidence between researchers and decision-makers, as well as aid in the creation and dissemination of larger bodies of evidence*	
**Tasks Relevant to Bridge Process**	**Bridge Process Examples**
1. Identify and obtain relevant information:	
Conduct environmental scan or needs assessment Define problem or research question Conduct evidence search and retrieval Connect stakeholders to relevant information sources Identify opportunities for integrating evidence into practice Identify implications for local programs, policies or practices	Identification, prioritization and refinement of priority health topics to research questions through a step-wise approach that includes an evidence search (Figure), engaging relevant stakeholders, and identifying opportunities to how the work may be integrated into practice (Stage 1-5)
2. Create tailored knowledge products:	
Prepare knowledge products and synthesis Tailor resources to stakeholder needs or local context	Development of lay evidence summaries for each priority health topic that is discussed at the Bridge Event (Stage 3) Support to tailor research findings to local context through MSSU Priority Project in-kind support
3. Project coordination:	
Provide administrative or research coordination support Support project funding proposals	Provide management, coordination and administrative support throughout Bridge Process (Stage 1-5) and on MSSU Priority Projects Support MSSU Priority Projects who would like to pursue further funding opportunities
**Linkage and Exchange Agent**^20^: *Positive relational skills and strategies, including skilled interpersonal, communication and adaptation skills, and strategies including networking, partnership development and collaboration across stakeholder groups*	
**Tasks Relevant to Bridge Process**	**Bridge Process Examples**
1. Identify, engage and connect stakeholders:	
Identify and engage relevant stakeholders Identify common goals among stakeholders Engage w/stakeholders in person	Identify and engage relevant stakeholders at an in-person event to discuss and align gaps and needs related to priority health issues (Stage 1-3)
2. Facilitate collaboration:	
Organize workshops or forums for collaboration Facilitate dialogue between stakeholders Facilitate consensus between stakeholders Facilitate relationship-building among stakeholders	Organization of the Bridge Event, follow-up meetings and networking opportunities to foster collaboration and relationships between stakeholder groups (Stage 3-5) Support information sharing and relationships through facilitated discussion and exercises at the Bridge Event and follow-up meetings (Stage 3-4)
3. Support communication and information sharing:	
Develop and maintain communication tools or strategies Communicate w/stakeholders Facilitate knowledge dissemination Support knowledge sharing among stakeholders	Development and implementation of communication/marketing materials and to promote Bridge Process including registration, press releases, targeted emails and presentations (Stage 2-3) Provide a forum for knowledge sharing and networking amongst stakeholder groups Support knowledge product development and dissemination for each MSSU Priority Project
4. Network, development, maintenance and facilitation:	
Identify networking opportunities for stakeholders	Provide networking opportunity through Bridge Event Aid in linking stakeholders across research, policy and practice setting during Bridge Process and post
5. Support sustainability	
Support the development of knowledge products Sustain engagement	Support and develop various knowledge products for each collaborative research partnership Help to sustain engagement of collaborative research partnerships by providing coordination and management support
**Capacity Builder**^20^: *Provide opportunities for researchers and decision-makers to strengthen skills in areas that may not be viewed traditional within their role, but vital to work between and with one another including communication, analytical and interpretive skills across sectors*	
**Tasks Relevant to Bridge Process**	**Bridge Process examples**
1. Facilitate development of analytic and interpretive skills:	
Design tailored training or educational sessions Seminars or workshops to enhance stakeholder skills Aid with interpretation of research Support peer-to-peer learning	Provide orientation of patient/citizen partners onto MSSU Priority Project teams and patient-oriented research training to team members Aid with interpretation of research including the development of evidence summaries related to each priority topic discussed at the Bridge Event (Stage 3), development of a glossary of health research terms to assist MSSU Priority Project team members

Abbreviation: MSSU, Maritime SPOR SUPPORT Unit.

####  Knowledge Management

*Knowledge Management* tasks related to the MSSU Bridge Process include: (1) Identify and obtain relevant information, (2) Create tailored knowledge products, and (3) Project coordination. As outlined in Table 3, these tasks span across various stages of the Bridge Process, with the most relevant tasks including identification, prioritization and refinement of priority health topics (Stages 1, 3), engaging stakeholders to integrate evidence into policy and practice support (Stages 1-5), development and adaption of knowledge products to stakeholder audiences through MSSU in-kind KT support (Stage 5), as well as management and coordination support offered to MSSU Priority Projects (Stages 4-5).

####  Linkage and Exchange Agents

*Linkage and Exchange Agents* tasks related to the Bridge Process include (1) Identify, engage and connect stakeholders, (2) Facilitate collaboration, (3) Support communication and information sharing. Most notably, Bridge Process tasks related to linkage and exchange include identifying and engaging relevant stakeholders to discuss and align gaps and needs related to priority health issues (Stage 1-3), organization of the Bridge Event, follow-up meetings and networking opportunities to foster collaboration and relationships between stakeholder groups (Stage 3-5), support information sharing through facilitated discussion at the Bridge Event, support collaborative dialogue through follow-up meetings (Stage 3-5), and aid in knowledge dissemination through MSSU in-kind support.

####  Capacity Builder

 Relevant *Capacity Builder* tasks implemented throughout the Bridge Process include activities outlined within the (1) Facilitate development of analytic and interpretive skills, including tailoring training or education sessions to stakeholders’ groups. Related Bridge Process tasks included orientation of patient/citizen partners to priority project teams, and the offering of patient-oriented research training to members of priority project teams. This orientation and training served as a way to introduce patient/citizen partners to MSSU procedures, processes, and staff. Additionally, training experiences allowed for opportunities to build knowledge related to patient-oriented health research. As well, the MSSU staff assist throughout the Bridge Process with interpretation of research findings and terminology including the development of evidence summaries related to each priority topic discussed at the Bridge Event (Stage 3), development of a glossary of health research terms to assist priority project team members, and ongoing assistance from MSSU staff in the adaption of knowledge products for an array of target audiences (Stage 5).

## Discussion

 This paper provided a detailed overview of the various components of an IKT approach by outlining the stages of the MSSU Bridge Process, the current quantitative and qualitative outputs, and outcomes, as well as the KB tasks conducted by the MSSU. Although there is growing evidence on the benefits of an IKT approach,^2,9^ as well as the barriers and enablers to the approach,^9,27^ there is limited research that presents strategies used in an IKT process in detail.^9,28^ This work aimed to contribute to the literature by providing actionable steps into the development, coordination and sustainment of this approach and the development collaborative research partnerships.

 Research that outlines the approach of IKT strategies is crucially needed to help guide professionals and practitioners on the, “how to” of IKT. Meetings, workshops, evidence briefs, web portals, consultations, deliberative dialogue and training sessions are all forms of IKT approaches,^9^ yet without detailed overview or evaluation, it can be difficult to use these as examples or inspiration in practice. Rycroft-Malone et al^29^ recent book, *Building Blocks for Research Coproduction *does provide useful steps of how to engage knowledge users in the research process, but real-world, in-depth examples such as the MSSU Bridge Process provides extra value of how this is done in practice. This gap is reiterated by Cardwell et al^30^ who placed effort in providing detailed methods, outcomes, and evaluation on their work using health hackathons – an innovative IKT approach that bring together diverse stakeholders to address complex health challenges.

 Beyond the local example of this real-world IKT process, this work provides insight into how this approach may be valuable, including establishment of meaningful connections and collaborations, focus on patient/citizen and stakeholder engagement, implications to policy and practice and being a catalyst for research funding. These findings are consistent with the literature that identifies various research and practical benefits of an IKT approach.^1,11-13^

 Of note, these benefits are also echoed in the patient engagement literature, suggesting that the involvement of patient/citizen partners leads to more applicable findings, research relevance and empowerment of knowledge users.^31-33^ However, our findings reiterate a common research thread suggesting that benefits of patient engagement do not come without the challenge of meaningful versus tokenistic engagement practices. Despite the commitment and intention for meaningful patient engagement across the Bridge Process, the feedback received from evaluation results suggests more work needs to be done to ensure patient/citizen voices are not only represented, but heard. Research environment, expectation, support, and value are four main themes highlighted in Black and colleagues’ qualitative study on what constitutes as meaningful patient engagement,^31^ yet practical barriers such as funding, time, compensation, recruitment and motivation, can often cause obstacles even when commitment and dedication are at the forefront.^34^

 This work also presents early outcomes of IKT partnerships including products developed from each collaborative research partnership (eg, reports, publications, infographics, etc), as well as examples of research, policy, and practice implications. However, aligned with past research, this work reiterates the challenge of measuring the attribution of an IKT approach to long-term health outcomes.^1,9,18,35^ This can be partially attributed to this study reporting on outcomes 1-2 years after initiation of the collaborative research partnerships, as well as the difficulty securing resources to evaluate the contribution of IKT to long-term health outcomes.

 In addition to the outputs of the Bridge Process, this work provided a detailed example of how MSSU as a KB played a critical role in the IKT approach. The research broke down the KB tasks into three domains guided by Bornbaum et al^20^: (1) *Knowledge Manager*, (2) *Linkage and Exchange Agent,* and (3) *Capacity Developer.* This indicated numerous responsibilities and affiliated tasks that are required for an IKT approach (eg, the Bridge Process), to be executed. Likewise to past work, the present work reiterated the multifaceted and often overlapping tasks that KBs may have when involved in a research partnership.^20,21,36^ Tasks associated with the domain of *Knowledge Manager* and *Linkage and Exchange *were the most prominent roles and responsibilities associated with the Bridge Process which is consistent with the responsibilities referenced in the literature.^21^ A key responsibility referenced for a *Knowledge Manager* was the ability to commission research through the identification of policy priority topics and aid in the transformation of these topics to clearly outlined research questions^21^ – a task particularly prominent in the Bridge Process. Also aligning with the literature, the main *Linkage and Exchange* tasks included crucial communication and adaptation skills, such as networking, partnership development and ability to collaborate across stakeholder groups.^20,37,38^ It should be emphasized that without the KB support offered by the MSSU many of these projects would not have come to fruition due to lack of financial and human resources.

###  Strengths and Limitations 

 This work highlighted valuable insight into the practical responsibilities, support and dedicated commitment needed to actionably facilitate the movement of evidence into practice. The MSSU was well positioned to support this work given its role as a KB within the community, which was facilitated by dedicated funding, support from local government and health authorities, and other research networks. Building and fostering these relationships takes dedicated resources and commitment within an organization. Further, the current research provides examples of utilizing an IKT approach across different health research areas, signifying the transferability of this process and affiliated skills needed for implementation. The work also outlines the assortment of pathways an IKT approach can unfold to inform practice in different ways including varying KT products, benefits and implications. However, with the opportunity for multiple branches of opportunities, an IKT approach can be iterative, non-linear and at times messy. These characteristics were evident throughout the Bridge Process and with that brought various limitations to the forefront.

 A notable limitation to this IKT approach is that due to the developmental nature of the Bridge Process, it was difficult to evaluate process and outcomes measures of the initiative. A prominent change of the Bridge Process included adaptation of *Stage 1: Identify priority health topics*. Various methods were tested to elicit priority topics from stakeholders in a timely manner including a survey and holding stakeholder meetings across partner organizations. Other adaptations in the process included logistics of the event such as timing, location, and structure, evolvement of the facilitator and patient/citizen partners role, and ongoing edits to the Bridge Event evaluation survey. Given the logistical challenges and changes to the survey across each Bridge Event, some potential outputs were difficult to report and/or could not be reported. Firstly, there were challenges in confirming the number of attendees for each event with some registrants being absent from the event or attending part of the event, and some attendees not registering prior leading to inconsistencies in registration and attendance information; therefore, we acknowledge this count as an estimate versus a concrete count of participants. Secondly, a low response rate to the evaluation surveys after each event, and further context around interpretation of some questions (eg, how participants interpreted ‘active engagement of patients’) may have provided clarity on outcomes to improve subsequent implementation efforts. Thirdly, in addition to the process, the collaborative research partnerships are all in different stages of the research process therefore it is difficult to report outcomes in unison. Notably, many of these partnerships are in their infancy and conducting pilot work that is projected to have larger impact. This is identified by the many partnerships that have been successful in securing further funding for future research initiatives. Lastly, the landscape in which the collaborative research is being developed and conducted cannot be overlooked. Changes in political management and team member commitment immensely impacts engagement in the research partnership, leading to ongoing onboarding of new members and difficulty in sustainment of partnerships over time.

###  Future Research 

 As noted by the limitations and past research, it is a challenge to measure the long-term health outcomes of an IKT approach.^2,39^ Kothari and Wathen^2^ suggest the next step in IKT research is to determine appropriate outcomes and impacts for evaluation including the meaning, value and effectiveness of having multi-stakeholder teams. Although this work shares known outputs and outcomes from the Bridge Process to date, future work needs to be conducted to follow up on the research and political implications of these collaborative research partnerships overtime. Additionally, it is important to evaluate the collaborative partnership model put in place to understand what components enable or mitigate these teams from sustaining and providing meaningful engagement opportunities for knowledge users and producers.^2,39^ Further, as a growing area in its own right, it may be of priority to specifically evaluate the patient/citizen partner involvement on the research teams to understand the unique obstacles this stakeholder group may face over time.

 In relation to KBs, more work needs to be done to clarify the roles and responsibilities of a KB and their necessity to drive research into policy and practice. There is potential value for KT researchers to explore the barriers and facilitators that KBs face when executing their roles in practice and what strategies can be put in place to mitigate common challenges and enhance common enablers. Current and past research has highlighted the vital support and coordination responsibilities a KB has, and action needs to take place to ensure these roles are valued, and further utilized within an IKT approach.

## Conclusion

 This work provided an overview of an IKT approach in action by outlining the practical stages of the Bridge Process, the diverse outputs and outcomes produced and the critical role MSSU played as a KB in the process. Although there are limitations to this work that can be attributed to the iterative process and infancy of some of the collaborative research partnerships discussed, this paper provides insight into the process and examples of how an IKT approach facilitates the closing of the “know-do gap” between research, policy and practice.

## Acknowledgements

 We would like to thank all MSSU team members, affiliated scientists and stakeholders who were involved in helping to organize and/or participate in different stages of the Bridge Process including patient/community partners, government personnel, health professionals, and the research community, within and across the Maritimes. We would also like to acknowledge each of the MSSU Priority Project teams including science and decision-maker leads, patient partners, and all other team members involved.

## Ethical issues

 This paper reports on the development and associated outcomes of an organizational strategy to facilitate research and evidence-informed decision-making. As this involves evaluation rather than research, no ethical approval was necessary based on the guidelines of Tri-Council Policy Statement: Ethical Conduct for Research Involving Humans.^40^

## Competing interests

 Authors declare that they have no competing interests.

## Authors’ contributions

 All authors contributed to the conception or design of the work, JK and AG contributed to the analysis and interpretation of the data. JK, AG, EJ, and LB drafted and revised the work. All authors read and approved the final manuscript. All authors agreed to be accountable for all aspects of the work.

## Funding

 This work was supported by the MSSU, which receives financial support from the Canadian Institute of Health Research (CIHR), the Nova Scotia Department of Health and Wellness, the New Brunswick Department of Health, the Nova Scotia Health Research Foundation, and the New Brunswick Health Research Foundation. The opinions, results and conclusions reported in this paper are those of the authors and are independent from the funding sources. No endorsement by the MSSU or the named funding partners is intended or should be inferred.

## Endnotes


^[1]^ The full name of the PUPPY study is Problems Coordinating and Accessing Primary Care for Attached and Unattached Patients Exacerbated During the COVID-19 Pandemic Year: A Longitudinal Mixed Methods Study With Rapid Reporting and Planning for the Road Ahead.

## Supplementary files


Supplementary file 1. MSSU Bridge Event Facilitator Guiding Document Example.

Supplementary file 2. MSSU Priority Project Application Example.

Supplementary file 3. MSSU Bridge Event Evaluation Survey Draft.

Supplementary file 4. MSSU Priority Projects Description, Stage, and Current Outputs With Available Links.
