# Supplementary material for: The Maritime SPOR SUPPORT Unit (MSSU) Bridge Process: An Integrated Knowledge Translation Approach to Address Priority Health Issues and Increase Collaborative Research in Nova Scotia, Canada
Source: Int J Health Policy Manag. 2023 Feb 14;12:6901. doi: 10.34172/ijhpm.2023.6901 (PMC10125170; doi:10.34172/ijhpm.2023.6901)
Supplement: Supplementary file 1 — MSSU Bridge Event Facilitator Guiding Document Example. [file ijhpm-12-6901-s001.pdf]

Article title: The Maritime SPOR SUPPORT Unit (MSSU) Bridge Process: An Integrated Knowledge Translation Approach to Address Priority Health Issues and Increase Collaborative Research in Nova Scotia, Canada

Journal name: International Journal of Health Policy and Management (IJHPM)

Authors' information: Julia Kontak[1]*, Amy Grant[1], Elizabeth Jeffers[1], Leah Boulos[1], Juanna Ricketts[1], Michael Davies[2], Marina Hamilton[1], Jill A. Hayden[3]

[1]Maritime SPOR SUPPORT Unit, Research and Innovation, Nova Scotia Health, Halifax, NS, Canada.

[2]Nova Scotia Department of Health and Wellness, Halifax, NS, Canada.

[3]Department of Community Health & Epidemiology, Faculty of Medicine, Dalhousie University, Halifax, NS, Canada.

(*Corresponding author: Julia.Kontak@dal.ca)

Supplementary file 1. MSSU Bridge Event Facilitator Guiding Document Example

Maritime SPOR SUPPORT Unit Bridge Event

Thank you for agreeing to be a facilitator for the Bridge Event. On the first page is an overview of the MSSU Bridge Event. One the second page is an overview of your roles and responsibilities as a facilitator.

MSSU Bridge Event

Aims:

1. Build connections and mobilize the knowledge we have in Nova Scotia from different perspectives, and
2. Identify knowledge gaps, enabling evidence-informed decision-making and improve healthcare and health outcomes in Nova Scotia.

Background:

The MSSU Bridge Event was established as a mechanism to bring together health policy decision makers, healthcare professionals, researchers, and patients/caregivers to discuss challenges, identify knowledge and evidence gaps, and consider potential solutions for specific priority-driven health issues.

Pre-Bridge Event:

Stage 1: Identification of priority health topics

The MSSU collaborates with the Department of Health and Wellness, Nova Scotia Health Authority and the IWK to obtain priority health topics where evidence gaps exist.

Stage 2: Identification of stakeholder expertise

Once priority health topics are defined, the MSSU utilizes its networks to engage health policy decision makers, healthcare professionals, researchers, patients, and caregivers with relevant expertise in these priority areas.

Day-of Bridge Event:

Stage 3: Identification of solutions to address priority topics

On the day of the Bridge Event, the newly formed teams engage in facilitated group discussions on the topic of interest and work together to identify knowledge gaps and potential solutions to address the issue(s). **The aim of the discussion is to narrow the focus, establish key research questions, and to create an action plan that will support the creation and implementation of a solution for the priority issue.**

Post-Bridge Event:

Stage 4: Developing and fostering collaborative research partnerships

Following the Bridge Event, the MSSU provides discussion summaries for the teams, and offers support to facilitate continued group interactions, carry out action plan activities, and foster interactions to enhance the early stages of team development.

Facilitation:

Overview:

As a facilitator for the MSSU Bridge Event you will be facilitating a group discussion on one of the five priority topics (outlined below). As a facilitator you will focus on the context and process of the discussion. A **facilitator package** that provides detailed information about the agenda has also been provided for you.

Roles & Responsibilities:

- Stay neutral on content
- Actively listen
- Synthesize and connect diverse perspectives
- Bring key ideas to the surface
- Notice biases
- Encourage all participants to contribute
- Keep discussion moving towards objective

Patient, Citizen, and Stakeholder Engagement:

The group that you will be facilitating will be made up of a diverse stakeholder including patient/citizens. It is generally expected that patients/citizens are drawing from **personal** health experience to contribute to discussion. This can involve drawing on challenging or life changing experiences. Important to keep this in mind; while everyone is expected to participate

constructively, emotions may still enter discussion. Balance being respectful with keeping discussion on track.

Technical Terms and Language:

As the groups will include varying types of stakeholders and diverse perspectives, please try to mitigate the use of technical terms and language by:

- Encouraging groups to avoid or explain technical terms
- Defining acronyms, write down on flipchart if possible
- Using clear, relevant examples
- Considering that participants may not ask for clarification
- Considering that group members may understand terms differently (e.g., PI = principal investigator and
