# Supplementary material for: The Maritime SPOR SUPPORT Unit (MSSU) Bridge Process: An Integrated Knowledge Translation Approach to Address Priority Health Issues and Increase Collaborative Research in Nova Scotia, Canada
Source: Int J Health Policy Manag. 2023 Feb 14;12:6901. doi: 10.34172/ijhpm.2023.6901 (PMC10125170; doi:10.34172/ijhpm.2023.6901)
Supplement: Supplementary file 2 — MSSU Priority Project Application Example. [file ijhpm-12-6901-s002.pdf]

Article title: The Maritime SPOR SUPPORT Unit (MSSU) Bridge Process: An Integrated Knowledge Translation Approach to Address Priority Health Issues and Increase Collaborative Research in Nova Scotia, Canada

Journal name: International Journal of Health Policy and Management (IJHPM)

Authors' information: Julia Kontak[1]*, Amy Grant[1], Elizabeth Jeffers[1], Leah Boulos[1], Juanna Ricketts[1], Michael Davies[2], Marina Hamilton[1], Jill A. Hayden[3]

[1]Maritime SPOR SUPPORT Unit, Research and Innovation, Nova Scotia Health, Halifax, NS, Canada.

[2]Nova Scotia Department of Health and Wellness, Halifax, NS, Canada.

[3]Department of Community Health & Epidemiology, Faculty of Medicine, Dalhousie University, Halifax, NS, Canada.

(*Corresponding author: Julia.Kontak@dal.ca)

Supplementary file 2. MSSU Priority Project Application Example

MSSU Nova Scotia Priority Project Application

This application is intended for research teams applying for MSSU Priority Project support in Nova Scotia. Research teams are typically formed through our bi-annual Nova Scotia Bridge Events.

Before applying, please read the MSSU NS Priority Project Application Guidelines. If you have questions or would like help preparing your application, please contact info@mssu.ca.

Project Details

Title

Research team

**Role**	**Name**	**Title, Affiliation**
Scientific Lead:		
Decision-Maker Lead(s):<br>*(NS Department of Health and Wellness, Nova Scotia Health Authority, and/or IWK Health*		

Centre)		
Patient/Citizen Partner(s):		
Additional Team Members:		

Project background and rationale (250 words)
Describe the health issue, existing evidence, and gap to be filled.

Research objective(s) (250 words)

Current stage of the project that you are undertaking:

☐ Team development
☐ Developing proposal
☐ Applying for funding
☐ Applying for REB and/or data access committee approval
☐ Conducting project
☐ Dissemination

Describe how this project fits into a larger program of research (if applicable), and any existing supports and funding (250 words)

How does the project align with MSSU key objectives? Check all that apply.

☐ The project is patient-oriented (for example, includes measures of outcomes important to patients, patient safety, or patient access to care)

☐ Has a plan for patient engagement or, in the case of a request for patient engagement support, a clear commitment to patient engagement

☐ The project will contribute to the implementation or evaluation of priority health system change in Nova Scotia

☐ The project involves new health data linkages

Describe alignment with MSSU key objectives (400 words)

Anticipated Outcomes and Impact

Project outputs (e.g., funding proposal, report, manuscript) (250 words)
Please outline the expected outputs during the period where you'd receive support from the MSSU.

Project implications (250 words)

In consultation with the research team's Decision-Maker Lead(s), describe the policy implications of this project. How will this research inform decision-making or change practice, impact the health system and patient care, and improve the health outcomes for Nova Scotians?

MSSU Project Support

In consultation with the MSSU, complete the table below to outline the role(s) of research team members and the MSSU support(s) requested for the project. Common activities are listed in a drop-down menu. If you need help with an activity not listed in the drop-down* menu, please specify using the 'other' option. Select as many activities as necessary; leave any additional fields blank.

**Listed activities in the drop-down menu include:* Proposal/protocol development Approaches to analysis, Literature searching or synthesis, REB assistance, HDNS data navigation and access, Database preparation, Data analysis, Presentation/manuscript preparation, Team communication, Project planning and coordination, Integration knowledge translation, Patient engagement, Contextualizing research, Communication and dissemination, Other.

Major Project Activities	Role of Research Team Members	MSSU Support Requested

E.g., Literature Search	*Scientific Lead will lead team discussions to determine research question and will work with the MSSU to develop search strategy.*	*Help fine-tuning research question, defining inclusion/exclusion criteria, developing search strategy, and running search.*
Select an activity		
Select an activity		
Select an activity		
Select an activity		

Project Timeline

Complete a timeline for the proposed period of support including the major and ongoing project activities from the table above.

Please note: NS Priority Projects can receive support for up to six months. If you require support over an extended period, please complete Appendix A.

Activity	Month 1	Month 2	Month 3	Month 4	Month 5	Month 6
	☐	☐	☐	☐	☐	☐
	☐	☐	☐	☐	☐	☐
	☐	☐	☐	☐	☐	☐
	☐	☐	☐	☐	☐	☐
	☐	☐	☐	☐	☐	☐
	☐	☐	☐	☐	☐	☐
	☐	☐	☐	☐	☐	☐
	☐	☐	☐	☐	☐	☐
	☐	☐	☐	☐	☐	☐
	☐	☐	☐	☐	☐	☐
	☐	☐	☐	☐	☐	☐
	☐	☐	☐	☐	☐	☐
	☐	☐	☐	☐	☐	☐
	☐	☐	☐	☐	☐	☐
	☐	☐	☐	☐	☐	☐
	☐	☐	☐	☐	☐	☐
	☐	☐	☐	☐	☐	☐

Signatures

Signature of Scientific Lead	Date	Signature of Decision-Maker Lead (1)	Date
Signature of Decision-Maker Lead (2), if applicable	Date	Signature of Decision-Maker Lead (3), if applicable	Date

Appendix A - Extended Project Timeline

If you require more than 6 months of support, please complete an additional timeline including the major and ongoing project activities.

Please note: Extensions for an additional six months may require an additional application, depending on research objectives.

Activity	Month 7	Month 8	Month 9	Month 10	Month 11	Month 12
	☐	☐	☐	☐	☐	☐
	☐	☐	☐	☐	☐	☐
	☐	☐	☐	☐	☐	☐
	☐	☐	☐	☐	☐	☐
	☐	☐	☐	☐	☐	☐
	☐	☐	☐	☐	☐	☐
	☐	☐	☐	☐	☐	☐
	☐	☐	☐	☐	☐	☐
	☐	☐	☐	☐	☐	☐
	☐	☐	☐	☐	☐	☐
	☐	☐	☐	☐	☐	☐
	☐	☐	☐	☐	☐	☐
	☐	☐	☐	☐	☐	☐
	☐	☐	☐	☐	☐	☐
	☐	☐	☐	☐	☐	☐
	☐	☐	☐	☐	☐	☐
	☐	☐	☐	☐	☐	☐
	☐	☐	☐	☐	☐	☐
	☐	☐	☐	☐	☐	☐
