# Supplementary material for: The Maritime SPOR SUPPORT Unit (MSSU) Bridge Process: An Integrated Knowledge Translation Approach to Address Priority Health Issues and Increase Collaborative Research in Nova Scotia, Canada
Source: Int J Health Policy Manag. 2023 Feb 14;12:6901. doi: 10.34172/ijhpm.2023.6901 (PMC10125170; doi:10.34172/ijhpm.2023.6901)
Supplement: Supplementary file 3 — MSSU Bridge Event Evaluation Survey Draft. [file ijhpm-12-6901-s003.pdf]

Article title: The Maritime SPOR SUPPORT Unit (MSSU) Bridge Process: An Integrated Knowledge Translation Approach to Address Priority Health Issues and Increase Collaborative Research in Nova Scotia, Canada

Journal name: International Journal of Health Policy and Management (IJHPM)

Authors' information: Julia Kontak[1]*, Amy Grant[1], Elizabeth Jeffers[1], Leah Boulos[1], Juanna Ricketts[1], Michael Davies[2], Marina Hamilton[1], Jill A. Hayden[3]

[1]Maritime SPOR SUPPORT Unit, Research and Innovation, Nova Scotia Health, Halifax, NS, Canada.

[2]Nova Scotia Department of Health and Wellness, Halifax, NS, Canada.

[3]Department of Community Health & Epidemiology, Faculty of Medicine, Dalhousie University, Halifax, NS, Canada.

(*Corresponding author: Julia.Kontak@dal.ca)

Supplementary file 3. MSSU Bridge Event Evaluation Survey Draft

MSSU Bridge Event Follow-up Survey:

Thank you for attending the MSSU Bridge Event. We would greatly appreciate if you could take the time to complete the following evaluation of this event:

General:

1. Which role best describes you?
   i. Researcher
   ii. Policy-maker
   iii. Healthcare provider
   iv. Patient, Caregiver, or Citizen
   v. Other
2. Which break-out session topic did you participate in?
3. How did you hear about this session?
   i. Email invitation
   ii. Referred by a colleague
   iii. Referred by MSSU staff
   iv. Other

Event Organization:

For questions 4-8 below, write and circle the appropriate number to the right of the text.	***1 Strongly Disagree***	***3 Neither Agree nor Disagree***	***5 Strongly Agree***
4. The organization of this event, with a one half-day session, was effective.			
5. There was enough time for discussion / individual participation in my group.			
6. The approach to the facilitated discussions was effective, as a means of identifying gaps and policy needs for a specific topic area.			
7. Patients/citizens were actively engaged and represented in the group discussion.			
8. There was enough time for networking with others during this event			

Event Venue:

For questions 9-10 below, write and circle the appropriate number to the right of the text.	***1 Strongly Disagree***	***3 Neither Agree nor Disagree***	***5 Strongly Agree***
9. The venue was satisfactory for the purpose of this event.			
10. The set-up of one large room was conducive to this event and the small group discussions.			

11. Please provide any further comments you may have regarding the venue below:

Insert your response here.

Event Objectives:

For question 12 below, write and circle the appropriate number to the right of the text.	***1 Strongly Disagree***	***3 Neither Agree nor Disagree***	***5 Strongly Agree***
12. As a result of this event:<br>i. I have engaged with researchers, healthcare providers, decision-makers, and/or patients/citizens I otherwise would not have met.<br>ii. I have a greater understanding of the gaps and policy needs in the specific topic areas presented today.			

Please consider the following statements and select the appropriate response.	***1 Strongly Disagree***	***3 Neither Agree nor Disagree***	***5 Strongly Agree***
13. The Bridge Event is an effective mechanism to initiate new collaborations/partnerships			
14. The Bridge Event is an effective mechanism to support health policy and/or practice change			

Please complete the following question to share your feedback on this event:

15. Overall evaluation of this session:

**Overall, I felt the day to be…**				
**Unsatisfactory**		**Average**		**Outstanding**
1	2	3	4	5

16. Would you attend a Bridge Event again?

Please explain why or why not.

Please explain why or why not.

17. Would you recommend this event to a colleague? Why or why not?

Please explain why or why not.

18. Please provide any general comments / feedback on this event.

Please answer here.
