# Supplementary material for: The Maritime SPOR SUPPORT Unit (MSSU) Bridge Process: An Integrated Knowledge Translation Approach to Address Priority Health Issues and Increase Collaborative Research in Nova Scotia, Canada
Source: Int J Health Policy Manag. 2023 Feb 14;12:6901. doi: 10.34172/ijhpm.2023.6901 (PMC10125170; doi:10.34172/ijhpm.2023.6901)
Supplement: Supplementary file 4 — MSSU Priority Projects Description, Stage, and Current Outputs With Available Links. [file ijhpm-12-6901-s004.pdf]

Article title: The Maritime SPOR SUPPORT Unit (MSSU) Bridge Process: An Integrated Knowledge Translation Approach to Address Priority Health Issues and Increase Collaborative Research in Nova Scotia, Canada

Journal name: International Journal of Health Policy and Management (IJHPM)

Authors' information: Julia Kontak[1]*, Amy Grant[1], Elizabeth Jeffers[1], Leah Boulos[1], Juanna Ricketts[1], Michael Davies[2], Marina Hamilton[1], Jill A. Hayden[3]

[1]Maritime SPOR SUPPORT Unit, Research and Innovation, Nova Scotia Health, Halifax, NS, Canada.

[2]Nova Scotia Department of Health and Wellness, Halifax, NS, Canada.

[3]Department of Community Health & Epidemiology, Faculty of Medicine, Dalhousie University, Halifax, NS, Canada.

(*Corresponding author: Julia.Kontak@dal.ca)

Supplementary file 4. MSSU Priority Projects Description, Stage, and Current Outputs With Available Links

	Project Title	Description of Project	Stage of Project	Deliverables
BP1 – June 2018	UniCITY: Uniting to connect innovative technology for youth mental health and addictions services<br><br>Part1: Scoping Review<br><br>Part 2: Asset Map	 <br><br><br><br>Part 1: A scoping review was conducted to examine the evidence-base for text-messaging in mental health and addictions services for children and youth.<br><br>Part 2: An asset map to capture and share information on eMental health programs, tools, resources, and services available to children and youth in Nova Scotia.	 <br><br><br><br>Part 1: Complete<br><br>Part 2: Complete	Peer-reviewed publication: JMIR Mental Health - Text Message Interventions in Adolescent Mental Health and Addiction Services: Scoping Review<br><br>Summary report: Participatory Asset Map – Summary Report<br><br>Full report: Participatory Asset Map<br><br>Successful funding for spin-off project: CIHR Catalyst Grant: Patient-Oriented Research ($99,445) - Seamless transitions for adolescent trajectories in care (STATIC)

	Barriers and enablers to implementing interprofessional collaborative family practice teams with a focus on improving access to primary care<br><br>Part 1: Literature review<br><br>Part 2: Survey	Part 1: A literature review was done to examine the barriers and enablers to interprofessional collaborative family practice teams, with focus on improving access to primary care.<br><br>Part 2: This project builds off the literature review to identify what barriers and enablers Collaborative Family Practice Teams in NS identify as important to their experience and which are of priority interest for remedial action.	Part 1: Complete<br><br>Part 2: In progress	Final report: Barriers and enablers to implementing interprofessional collaborative family practice teams with a focus on improving access to primary care: A review of the literature<br><br>Infographic: Barriers and enablers to implementing interprofessional collaborative family practice teams<br><br>Conference presentation: Canadian Association for Health Services and Policy Research (CAHSPR) 2020 poster - Barriers and Enablers to Implementing Interprofessional Collaborative Family Practice Teams with a Focus on Improving Access to Primary Care<br><br>Conference presentation: BRIC NS Virtual Poster Day - Barriers and Enablers to Implementing Interprofessional Collaborative Family Practice Teams with a Focus on Improving Access to Primary Care
	Current management and healthcare quality for patients with hip and knee osteoarthritis	This study aims to better understand current functioning of individuals with osteoarthritis in the three Maritime Provinces and to examine healthcare utilization patterns of individuals with osteoarthritis in Nova Scotia using administrative healthcare data.	In progress	Conference presentation (abstract published): Osteoarthritis Research Society International<br><br>Successful funding for spin-off project: Research Nova Scotia Development/Innovative Grant ($15,000) - Current Management and Health Care Quality for Patients with Knee and Hip Osteoarthritis

BP 2 – November 2018	Pharmacist prescribing and primary healthcare access<br><br>Part 1: Administrative data project<br><br>Part 2: Pharmacist survey	Part 1: This study aimed to describe the characteristics of pharmacist prescribers and prescribing activities, as well as patient characteristics for those who use the services of pharmacist prescribers, using the Drug Information System.<br><br>Part 2: This study aimed to describe pharmacist prescribing behaviour, including extent of prescribing, perceptions of their prescribing role, barriers and facilitators to prescribing, and changes over time, through self-reported data.	Part 1: Complete<br><br>Part 2: Complete	Peer-reviewed publication: Increased self-reported pharmacist prescribing during the COVID-19 pandemic: Using the Theoretical Domains Framework to identify barriers and facilitators to prescribing<br><br>Canadian Association for Health Services and Policy Research (CAHSPR) oral presentations:<br><br>Pharmacist Prescribing in Nova Scotia: A Qualitative Analysis of Self-Perceived Barriers<br><br>Barriers and facilitators to Nova Scotia pharmacist's self-reported prescribing practices: A quantitative analysis<br><br>Pharmacist Prescribing and Primary Healthcare Access<br><br>Successful funding and spin-off project: CIHR Operating Grant COVID-19 Rapid Research ($407,552) - PUPPY Study - Problems Coordinating and Accessing Primary Care for Attached and Unattached Patients Exacerbated During the COVID-19 Pandemic Year: A Longitudinal Mixed Methods Study with Rapid Reporting and Planning for the Road Ahead
BP 3 – June 2019	Youth and young adult vaping: Gathering evidence and guiding practice	Development of educational resources for teachers and youth based on survey results, examining the types of vapers, reasons for use, mechanisms explaining use and moderators of use in Nova Scotia.	Complete	Infographics:<br><br>Vaping in Youth: A tip sheet for Health Professionals<br><br>Vaping in Youth: What do you need to know?

	Exploring the transition from pediatric to adult care<br><br>Part 1: Healthcare service utilization<br><br>Part 2: Qualitative data collection	Part 1: This study will utilize health administrative data to describe patterns of healthcare utilization for youth with chronic disease (IBD, Diabetes, CF, JIA) before and after transfer to adult care and to identify predictors (e.g., age of diagnosis, area of residence, continuity of primary care, number of pediatric visits pre-transfer, length of time between visit to pediatric to adult provider) of high- and low-quality care transfer.<br><br>Part 2: This study described the experiences of and barriers/enablers to transition amongst pediatric patients currently in the process of transitioning from pediatric to adult care, and former pediatric patients who have transferred to adult care and their caregivers, as well as describe clinicians' perspective on process and barriers/enablers to preparing patients and families for the transition from pediatric to adult care.	Part 1: In progress<br><br>Part 2: Complete	Peer-reviewed publication: Provider perspectives of barriers and facilitators to the transition from pediatric to adult care: a qualitative descriptive study using the COM-B model of behaviour<br><br>Summary report: The barriers and facilitators of transition from pediatric to adult health care in Nova Scotia<br><br>Conference presentation: Children's Healthcare Canada Transitions to Adulthood Pop-up Event – Exploring the Transition from Pediatric to Adult Health Care<br><br>Conference presentation: Canadian Association for Health Services and Policy Research (CAHSPR) oral presentation: Using an Integrated Knowledge Translation Approach to Explore the Transition from Pediatric to Adult Health Care in Nova Scotia<br><br>Infographics:<br>Transition of Care Infographics

BP 4 – November 2019	How does Nova Scotia best harness the assets of its extensive post-secondary educational system to improve regional health outcomes?<br><br>Part 1: Scoping Review<br><br>Part 2: Qualitative Data Collection	A comprehensive synthesis of evidence to support the implementation of learning health systems (LHS) to guide organizations in the effective and sustained implementation of such systems in Nova Scotia.<br><br>Part 1: Identify existing models of organizational partnership aimed at facilitating knowledge generation and exchange that are relevant to promoting LHS in the Nova Scotia context, through conducting a scoping review.<br><br>Part 2: Explore barriers and facilitators to LHS in the Nova Scotia context in order to identify opportunities and strategies to support a provincial LHS, using qualitative interviews and focus groups with key leaders and representatives from academic and health systems.	Part 1: In progress<br><br>Part 2: Not started	Conference presentation: Promoting Health through Collaborative Research and Innovation in Nova Scotia's Eastern Zone (Hosted by NS Health and Cape Breton University): Academic and Health Authority Partnerships – A Comprehensive Scoping Review

Note: Table and hyperlinks are up to date as of September 29th, 2022
